# Effects of Postprandial Lipemia Combined With Disturbed Blood Flow on the Flow-Mediated Dilation, Oxidative Stress, and Endothelial Microvesicles in Healthy Subjects

**DOI:** 10.3389/fphys.2022.812942

**Published:** 2022-02-24

**Authors:** Gustavo S. M. Araujo, Thiago O. C. Silva, Grazia M. Guerra, João E. Izaias, Helena M. N. Rocha, Diego Faria, Natalia G. Rocha, Aline Lopes Dalmazo, Amanda Araujo, Fernanda Marciano Consolim-Colombo, Katia de Angelis, Maria C. C. Irigoyen, Allan R. K. Sales

**Affiliations:** ^1^Heart Institute, University of São Paulo Medical School, Universidade Nove de Julho (UNINOVE), São Paulo, Brazil; ^2^D’OR Institute for Research and Education, São Paulo, Brazil; ^3^Department of Physiology and Pharmacology, Fluminense Federal University, Niterói, Brazil; ^4^Cardiology Institute of Rio Grande do Sul/Cardiology University Foundation (IC-FUC), Porto Alegre, Brazil; ^5^Department of Physiology, Federal University of São Paulo, São Paulo, Brazil

**Keywords:** disturbance in blood flow patterns, postprandial lipemia, endothelial function, oxidative stress, endothelial microvesicles

## Abstract

**Aims:**

Both postprandial lipemia (PPL) and disturbed blood flow (DBF) induce endothelial dysfunction. However, the interactive effect of these stimuli on endothelial function is currently unknown. In the present study, we tested whether PPL plus DBF causes a greater reduction in flow-mediated dilation (FMD) than PPL and if this response is associated with elevations in oxidative stress and endothelial microvesicles (EMVs).

**Methods:**

Eighteen individuals (aged 28 ± 1yrs, 3 females, and BMI 24.43 ± 0.8kg/m^2^) randomly underwent two experimental sessions: PPL and PPL plus DBF. FMD and venous blood samples were obtained at baseline and 30, 70, and 110 min after stimulation. PPL was induced by fat overload *via* mozzarella pizza ingestion and DBF by forearm cuff inflation to 75 mm Hg per 30 min. Lipidic profile, oxidative stress (thiobarbituric acid reactive substances, TBARS; ferric reducing/antioxidant power, FRAP; hydrogen peroxide, H_2_O_2_) and EMVs were measured in blood samples.

**Results:**

Hypertriglyceridemia was observed in both sessions. Retrograde shear rate and oscillatory index responses were significantly higher in the PPL plus DBF compared with PPL. PPL plus DBF evoked a greater reduction in FMD than did PPL and EMVs, NADPH oxidase, and H_2_O_2_ similarly increased in both sessions, but TBARS and FRAP did not change.

**Conclusion:**

These data indicate that the association of PPL plus DBF additively impairs endothelium-dependent function in 110 min after stimulus in healthy individuals, despite a similar increase in oxidative stress and EMVs. Further studies are needed to understand the mechanisms associated with the induced-endothelial dysfunction by association of PPL and DBF.

## Introduction

Coronary arterial disease is the main cause of death and reduction in quality of life worldwide, leading to enormous economic consequences (Mathers and Loncar, 2006; Stone et al., 2012). Atherosclerosis is a complex process and evolves several stages, in which endothelial dysfunction has a central role (Stone et al., 2012; Gimbrone and García-Cardeña, 2016).

An important risk factor for endothelial dysfunction is postprandial lipidemia (PPL) (Nordestgaard et al., 2007; Nakamura et al., 2016; Zhao et al., 2021). The ingestion of a high-fat meal increases serum triglyceride concentrations, induces systemic oxidative stress, and impairs flow-mediated dilation (FMD) in both healthy subjects and patients with dyslipidemia (Tsai et al., 2004; Cortés et al., 2006; Ramírez-Vélez, 2011; Petersen et al., 2020). Postprandial lipidemia-induced vascular endothelial dysfunction is thought to be mediated by oxidative stress resulting from an increased lipid load within the cell, in turn leading to increased oxidative metabolism and excess production of reactive oxygen species (ROS) (Ramírez-Vélez, 2011; Zhao et al., 2021).

It is well known that geometrically irregular arterial regions including bifurcations, branches, and curvatures, which are characterized by low time-average shear stress and high oscillatory blood flow profiles (i.e., disturbed blood flow, DBF), are markedly susceptible to the development of atherosclerosis (Chiu and Chien, 2011; Davies et al., 2013). These findings are supported by cell culture and isolated vessel studies that demonstrate that endothelial injury and pathologic vascular remodeling are induced by DBF. Low shear rate (SR) reduces NO bioavailability by decreasing eNOS expression, thereby exposing the endothelium to the atherogenic effect of risk factors. In humans, it has been consistently shown that acute elevations to retrograde and oscillatory SR decrease the FMD of atherosclerosis-prone and resistant conduct arteries (Thijssen et al., 2009; Schreuder et al., 2014; Silva et al., 2021). However, the interactive effect of blood flow disturbances and PPL on endothelial function is currently unknown.

Endothelial microvesicles (EMVs) are small vesicles released from the endothelium into the circulation in response to activation, injury, or apoptosis of endothelial cells (Dignat-George and Boulanger, 2011). They have been systemically and clinically used as a marker of both cardiovascular disease (Schiro et al., 2014) and risk factors (Berezin et al., 2015). Previous evidence showed that a single high-fat meal produced a significant increase of EMV plasma levels in healthy normolipidemic individuals (Ferreira et al., 2004; Tushuizen et al., 2006). Also, acute elevations in retrograde and oscillatory SR on the brachial artery increases EMV levels in the circulation of healthy subjects (Whittaker and Przyklenk, n.d.; Jenkins et al., 2013) and patients with risk factors (Rocha et al., 2018) and cardiovascular disease (Silva et al., 2021). However, whether the combination of PPL and DBF exacerbate the increase in EMV levels is also unknown.

In the present study, we hypothesized that short-term vascular exposure to PPL and DBF additively impairs endothelial function, as assessed by FMD, in young healthy subjects. We also attempted to determine whether this additive effect in the reduction of FMD induced by PPL and DBF is associated with increases in both circulating oxidative stress and EMVs.

## Materials and Methods

### Ethical Approval

All experimental procedures and measurements conformed to the Declaration of Helsinki and were approved by the Research Committee of the Heart Institute (SDC 4549/17/049), the Human Subject Protection Committee at the Clinics Hospital of the University of São Paulo Medical School (CAAE 71981517.8.0000.0068). All participants provided written informed consent before enrollment.

### Samples

A total of 18 healthy subjects, non-smoking, recreational to highly active (endurance exercise ≥ 3 h week^–1^), aged 18–35 years, with no history of cardiovascular or metabolic diseases were included in this study. Initially, the participants were screened by telephone and reported to the laboratory following an overnight fast for a screening visit to verify eligibility. Exclusion criteria were as follows: systolic blood pressure ≥ 140 mm Hg, diastolic blood pressure ≥ 90 mm Hg, serum total cholesterol ≥ 200 mg dL^–1^; low-density lipoprotein-cholesterol ≥ 130 mg dL^–1^; high-density lipoprotein-cholesterol ≤ 35 mg dL^–1^; fasting glucose ≥ 100 mg dL^–1^. Screening for the study consisted of a complete medical history questionnaire, followed by a physical examination that included biochemical blood analysis, resting blood pressure (BP) measurement (sphygmomanometry), a resting electrocardiogram, and anthropometry (i.e., body weight and height).

### Experimental Design

**FIGURE 1:**
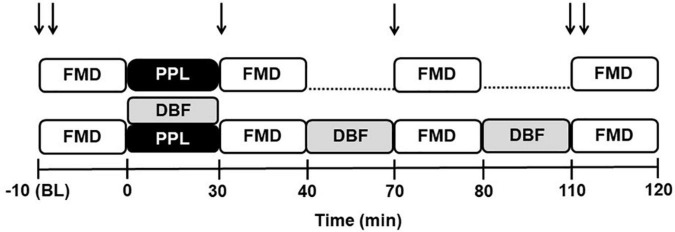
Experimental design for postprandial lipemia (PPL) and PPL plus disturbed blood flow (DBF) sessions. FMD, brachial artery flow-mediated dilation; ↓↓, oxidative stress, endothelial microvesicles and lipidic profile measurements; ↓, glucose and lipidic profile.

The study protocol was performed randomly in two sessions, PPL and PPL plus DBF (Figure 1). All sessions were conducted in the morning and after 12-h fast, seven days apart. Before each visit, subjects abstained from alcohol and caffeine for 24 h and from intense physical activity for 48 h. In the PPL session, the participants provided venous blood samples to measure total cholesterol and fractions, triglycerides, glucose, oxidative stress markers, and circulating EMVs, followed by evaluation of the endothelium-dependent function of the brachial artery, *via* FMD. Then, the participants underwent a fat overload, *via* ingestion of a mozzarella pizza. At 30, 70, and 110 min after the fat overload, venous blood samples were collected to measure total cholesterol and fractions, triglycerides, glucose, oxidative stress markers, and circulating EMVs and FMD. The PPL plus DBF session followed the same protocol of PPL, but the participants also underwent blood flow disturbance maneuvers for 30 min at three time points (0, 40, and 80 min).

### Measures and Procedures

#### Flow Mediated Dilation

Brachial artery FMD was measured in the left arm, with the shoulder abducted at approximately 80° and the forearm supinated. In accordance with the most recent FMD guidelines (Thijssen et al., 2011, 2019), an appropriate-sized rapid inflation/deflation pneumatic cuff (E-20 Rapid Cuff Inflator, D.E. Hokanson) was placed around the left forearm, immediately distal to the olecranon process. Brachial images (2–12 cm above the antecubital fossa) were obtained through duplex mode and ultrasound equipment (Vivid E9, General Electric, Horten, Norway) equipped with a 13 MHz linear probe. The contrast resolution, depth, and gain were adjusted to optimize the longitudinal images of the lumen/arterial wall interface. Brachial artery diameter and insonation angle-corrected (≤ 60°) blood velocity spectra were simultaneously recorded *via* the pulsed-wave mode at linear frequencies of 13 and 6.0 MHz, respectively. The sample volume was located at the center of the brachial artery and then adjusted to cover vessel width. After this, baseline diameter and blood velocity waveforms were continuously recorded over 60 s. Reactive hyperemia was assessed immediately after relief of 5 min of total ischemia, which was induced by external compression of the arm using an inflatable cuff at 220 mm Hg. After this period, the cuff was rapidly deflated and the changes in artery diameters from baseline were expressed as percentages. The probe location was marked on the skin to guarantee that the FMD was repeated in the same location during the study.

#### Postprandial Lipemia

Postprandial lipidemia (PPL) was induced through the ingestion of a mozzarella pizza (Sadia), with a total of 460 g and the following nutritional information: 59.4 g of fat, 102 g of carbohydrates, and 72 g of protein. The total energy value of this pizza is 1230Kcal. The pizza was divided into 6 pieces and participants had to consume each portion in 5 min during a total period of 30 min. Previous published and unpublished data (pilot study) have demonstrated that this fat overload model efficiently reduces the endothelium-dependent function of the brachial artery (Tyldum et al., 2009).

#### Disturbed Blood Flow Intervention

After 10 min of quiet resting, a 30-min intervention to induce DBF or control) was conducted at three time points (0, 40, and 80 min). Then, a pneumatic cuff (Hokanson) was placed around the left forearm and inflated to 75 mm Hg, and a second proximal cuff was positioned 3 cm distal from the axilla and inflated to 40 mm Hg to partially occlude venous flow from the arm. For normal flow measures, the cuff was placed on the same arm, but it was not inflated. Mean SR and the pattern of SR (anterograde, retrograde, and oscillatory) were recorded by 60 s at three different time points, 0 (before the inflation of the cuffs), and 15 and 30 min (during inflation of the cuffs).

#### Oxidative Stress

Whole blood was sampled from the participants in EDTA tubes and then centrifuged at 448 relative centrifugal force (rcp) for 10 min at 4°C. Plasma was removed and kept aside for further analysis. The supernatant was stored at −80°C for subsequent assessments. Proteins were quantified by the method described by Oliver et al. (1951).

#### Nicotinamide Adenine Dinucleotide Phosphate Oxidase

Nicotinamide adenine dinucleotide phosphate oxidase (NADPH) oxidase was determined by the rate of NADPH consumption assessed by measuring the decline in absorbance (340 nm) every 10 min, using a plate reader spectrophotometer (Espectra Max 2, Molecular Devices) (Wei et al., 2006). For the assay, we used a 50 mM phosphate buffer containing EDTA (2 mM, Nuclear, 311737), sucrose (150 mM, Sigma–Aldrich Corporation, S7903), NADPH (1.3 mM, Sigma–Aldrich Corporation, N1630), and 10 μL of sample.

#### Hydrogen Peroxide

Hydrogen Peroxide (H_2_O_2_) was analyzed by measuring oxidation of phenol red (Sigma–Aldrich Corporation, H3410) mediated by radish peroxidase (Sigma–Aldrich Corporation, P8250), using a plate reader spectrophotometer (610 nm, Espectra Max 2, Molecular Devices) (Pick and Keisari, 1980).

#### Lipoperoxidation

Plasma lipid peroxide levels were determined by measuring thiobarbituric acid reactive substances (TBARSs), a common method for measuring the concentration of malondialdehyde, the main breakdown product of oxidized lipids. For the TBARS assay, using 250 μL of sample, trichloroacetic acid (10%, wt/vol, Dinamica, 1072-1) was added to the homogenate to precipitate proteins and to acidify the samples. This mixture was then centrifuged (1792 rcp, 10 min), the protein-free sample was extracted, and thiobarbituric acid (0.67%, wt/vol, Sigma–Aldrich Corporation, T-550-0) was added to the reaction medium. The tubes were placed in a water bath (100°C) for 30 min. The absorbances were measured at 535 nm using a spectrophotometer (SP22, Bioespectro) (Buege and Aust, 1978).

### Ferric Reducing/Antioxidant Power

Total antioxidant activity was determined using the ferric reducing/antioxidant power (FRAP) assay (Benzie and Strain, 1999). Sodium acetate and acetic acid buffer solution were mixed with a standard solution of ferrous sulfate heptahydrate or sample stored in microplates. The microplates were measured spectrophotometrically at 593 nm. The value was expressed in mM of Fe (II).

#### Endothelial Microvesicles

Venous blood samples were collected in vials containing acid citrate dextrose before (baseline) and during [-10 (BL) and 110 min, respectively] experimental and control sessions. The endothelial microparticle populations CD31^+^/CD42b^–^ were determined by flow cytometry (BD FACS Verse; BD Biosciences; Franklin Lakes, NJ, United States), as previously described (Rocha et al., 2018; Toschi-Dias et al., 2021). Briefly, 50μL of platelet-poor plasma samples were incubated in the dark with 4μL of CD31-FITC (BD Biosciences; Franklin Lakes, NJ, United States) and 4 μL of CD42b- APC (BD Biosciences; Franklin Lakes, NJ, United States) for 30 min at 4°C. Samples were diluted with 450 μL of sterile PBS before flow cytometry analysis. Microvesicles were determined as events smaller than 0.9 μm (0.9 μm NIST Traceable polystyrene particle beads, Polysciences Inc., Warrington, PA, United States). Flow rate was set on low, and all samples were run for 90 s. TruCount beads (BD Biosciences; Franklin Lakes, NJ, United States) were used to calculate the concentration of microvesicles by using (microvesicles per microliter of plasma) the following formula according to manufacturer’s instructions: [(number of events acquired/absolute number of TruCount beads) X (total number of TruCount beads per test/total sample volume)].

### Data Analysis

The video files were compatible with commercial automated edge-detection and wall-tracking software (Cardiovascular Suite, Pisa, Italy), which was used for offline analysis. The initial phase of the software analysis consisted of identifying regions of interest on the optimal portion of the brachial artery image and its blood velocity spectra. R-wave gaiting function was applied to continuously assess brachial artery diameter or blood velocity. Flow-mediated dilation was calculated as the percentage rise of this peak diameter from the preceding baseline diameter. After deflation of the cuff, the cumulative shear rate was determined by a 3-min period through the area under the curve (AUC; s^–1^⋅). But the FMD was normalized by SR-AUC until peak vasodilation (peak diameter). FMD reliability was evaluated through the comparison of FMD value (pre-intervention) between days. The intraclass correlation coefficient was 0.93 (*p* < 0.05), and the coefficient of variation was 4.5%.

Shear rate (SR) was calculated as four times the ratio between mean blood velocity (*V*_mean_; in cm/s) and artery diameter (in cm) [i.e., SR = 4 × (*V*_mean_/diameter)]. For calculations of antegrade and retrograde SR, antegrade and retrograde mean blood velocities were used, respectively. In addition, oscillatory SR index, a variable that estimates the magnitude of oscillation in the vascular bed was calculated as IRetrograde shearI/IAntegrade shearI + IRetrograde shearI. Oscillatory SR index values range from 0 to 0.5, with 0 corresponding to unidirectional shear throughout the cardiac cycle, and 0.5 representing pure oscillation with time-average shear equal to 0. Oscillatory SR was expressed in arbitrary units (a.u.). The FMD, oxidative stress and EMPs response to experimental sessions is presented as—10 min (baseline) and time point 110 min. The absolute changes (delta) were calculated by subtracting values at 110 min from baseline.

### Statistical Analysis

The Shapiro-Wilk test was used to verify data distribution, and the Mauchly test was used to verify sphericity. The normality and sphericity assumptions were not violated. The Student paired *t*-test was used to compare differences between deltas. Two-way repeated-measures ANOVA were used to analyze FMD variables, SR patterns, lipidic and glycemic profiles, oxidative stress, and EMVs between LPP and LPP plus DBF sessions. Fisher LSD *post hoc* tests were applied when significant *F* values were found. Data are presented as mean ± standard deviation, percentage, or absolute delta. Significance was set at *p* ≤ 0.05. All analyses were performed using STATISITIC 12.0 software.

## Results

### Physical and Clinical Characteristics

The physical and clinical characteristics of the participants are presented in Table 1. Participants did not present obesity, arterial hypertension, elevated resting heart rate, and alterations in both lipid and glycemic profiles.

**TABLE 1:** Baseline characteristics.

Variables	
*N*	18
Sex, female/male	15/3
Age, yrs	27.0 ± 4.0
Weight	76.8 ± 11.6
Height, cm	175.0 ± 8.0
BMI, Kg/m^2^	24.9 ± 2.8
Systolic BP, mm Hg	116.0 ± 10.0
Diastolic BP, mm Hg	68.0 ± 6.0
Mean BP, mm Hg	86.0 ± 6.0
HR, bpm	73.0 ± 10.0
Triglycerides, mg/dl	80.6 ± 42.0
Total Cholesterol, mg/dl	160.8 ± 33.7
LDL Cholesterol, mg/dl	94.2 ± 30.5
HDL Cholesterol, mg/dl	48.2 ± 11.0
Glucose, mg/dl	88.0 ± 1.6

*Data presented as standard deviation. BMI, Body mass index; Systolic BP, Systolic blood pressure; Diastolic BP, diastolic blood pressure; Mean BP, mean blood pressure; HR, Heart rate; LDL Cholesterol, low-density lipoprotein and HDL Cholesterol, High-density lipoprotein.*

### Metabolic Response to Fat Load

**FIGURE 2:**
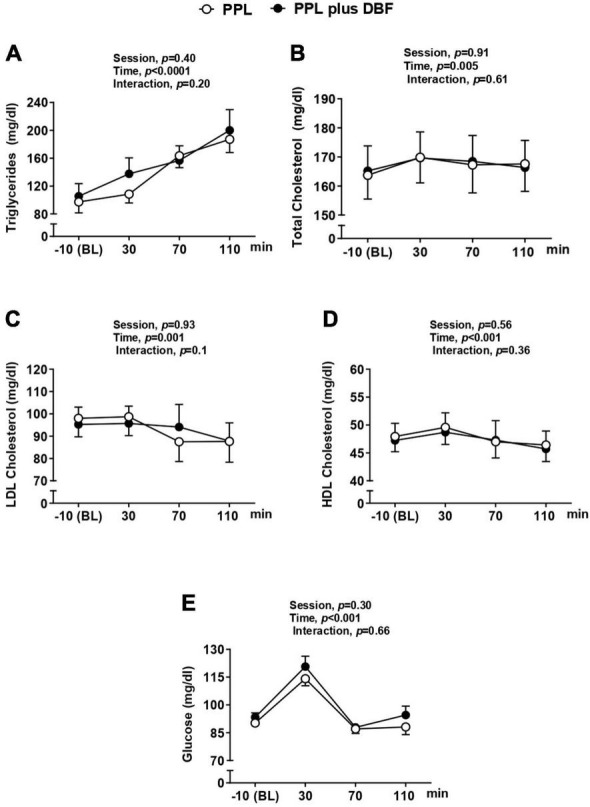
Triglycerides **(A)**, glucose **(B)**, total cholesterol **(C)**, and HDL cholesterol **(D)** to postprandial lipemia (PPL) and PPL plus disturbed blood flow (DBF) in healthy individuals. HDL cholesterol, high density lipoprotein cholesterol. Data presented as mean ± standard deviation.

The metabolic responses to fat load in both PPL and PPL plus DBF sessions are displayed in the Figures 2A–E. The ingestion of pizza similarly increased the circulating fasting levels of triglycerides (A Panel), total cholesterol (B Panel), LDL cholesterol (C Panel), HDL cholesterol (D Panel), and glucose (E Panel) in both sessions (Time effect *p* < 0.05 to all variables). These results clearly show that our model, *via* ingestion of mozzarella pizza, was able to induce systemic fat load.

### Disturbed Blood Flow

**FIGURE 3:**
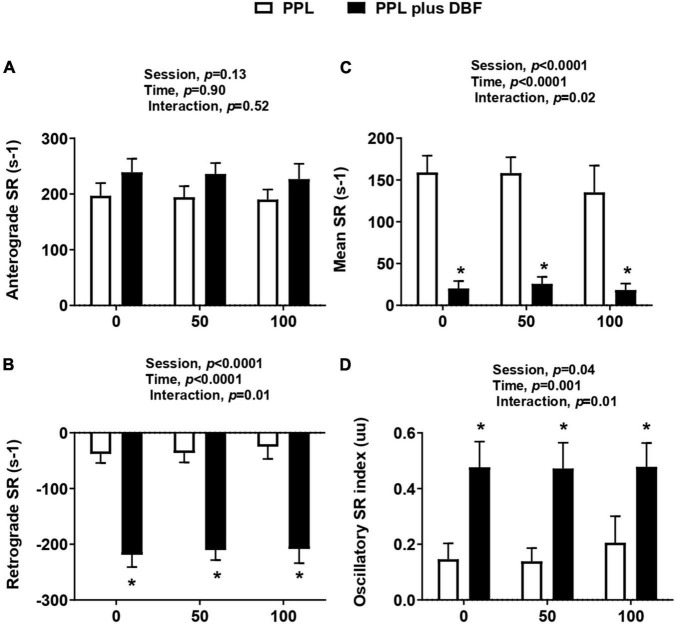
Anterograde SR **(A)**, retrograde SR **(B)**, mean SR **(C)**, and oscillatory index **(D)** to postprandial lipemia (PPL) and PPL plus disturbed blood flow (DBF) in healthy individuals. Anterograde SR, anterograde shear rate; retrograde SR, retrograde shear rate; mean SR, mean shear rate; oscillatory SR, oscillatory shear rate. Data presented as mean ± standard deviation. Absolute delta was calculated as peak variable–baseline variable. * *p* < 0.05 differences between sessions.

As expected, retrograde SR and oscillatory index responses to forearm cuff inflation at the three time points (0, 40, and 70 min) were greater in PPL plus DBF compared with PPL (interaction effect, *p* < 0.05, to both variables, Figures 3B–D, respectively). Mean SR response was significantly smaller in PPL plus DBF compared with PPL (interaction effect, *p* < 0.05, Figure 3C). Antegrade SR was not different between sessions (interaction effect, *p* > 0.05, Figure 3A).

### Flow Mediated Dilation to Postprandial Lipemia Plus Disturbed Blood Flow

**FIGURE 4:**
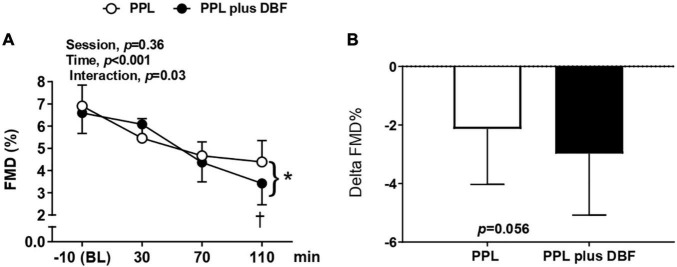
Brachial artery flow-mediated dilation [FMD, **(A)**] and delta FMD **(B)** to postprandial lipemia (PPL) and PPL plus disturbed blood flow (DBF) in healthy individuals. Data presented as mean ± standard deviation. * *p* < 0.05 vs. −10 min (BL) in both sessions. ^†^ *p* < 0.05 differences between sessions in time point 110 min. Absolute delta was calculated as 110 min FMD–baseline FMD.

Our analysis revealed that both PPL and PPL plus DBF decreased the brachial artery FMD% at the time points (30, 70, and 110 min) relative to basal (-10 min) (interaction effect, *p* = 0.03, Figure 4A), but this reduction was greater at the 110 min time point of PPL plus DBF (*p* < 0.05). Delta analysis showed that brachial artery FMD% was decreased to a greater extent (at 110min) with addition of DBF (*p* = 0.056, Figure 4B).

Our data revealed that both PPL plus DBF and PPL plus DBF did not change the resting diameter, peak diameter, SR-AUC, and time to peak (interaction effect, *p* > 0.05 to all variables, Table 2). PPL and PPL plus DBF decreased the brachial artery FMD (mm) at the time points (30, 70, and 110 min) relative to basal (-10 min) (interaction effect, *p* = 0.04, Table 2), but this reduction was greater at the 110 min time point of PPL plus DBF (*p* = 0.03).

**TABLE 2:** Vascular variables of flow-mediated dilation during postprandial lipemia and postprandial lipemia plus disturbed blood flow.

Variables	−10 (BL)	30	70	110 min
Rest diameter, mm				
PPL	3.54 ± 0.62	3.52 ± 0.63	3.54 ± 0.61	3.54 ± 0.61
PPL plus DBF	3.60 ± 0.54	3.58 ± 0.48	3.58 ± 0.54	3.59 ± 0.55
Peak diameter, mm				
PPL	3.77 ± 0.68	3.71 ± 0.66	3.70 ± 0.62	3.70 ± 0.62
PPL plus DBF	3.83 ± 0.57	3.77 ± 0.49	3.73 ± 0.53	3.71 ± 0.56
FMD, mm				
PPL	0.24 ± 0.08	0.19 ± 0.06*	0.16 ± 0.04*	0.15 ± 0.06*
PPL plus DBF	0.24 ± 0.07	0.21 ± 0.09*	0.15 ± 0.05*	0.11 ± 0.05*^†^
SR AUC10^–5^,_S_^–1^				
PPL	2.47 ± 1.20	14.23 ± 1.19	2.94 ± 1.34	2.69 ± 1.18
PPL plus DBF	2.53 ± 1.23	2.65 ± 1.31	2.53 ± 1.30	2.71 ± 1.38
Time to peak, seg				
PPL	55.70 ± 23.12	81.46 ± 32.35	66.80 ± 30.14	55.80 ± 23.13
PPL plus DBF	58.73 ± 32.21	62.46 ± 32.14	56.00 ± 25.22	73.80 ± 44.02

Data are presented as mean ± standard deviation. PPL, Postprandial lipemia; PPL plus DBF, Postprandial lipemia plus disturbed blood flow; FMD, Flow-mediated dilation and SRAUC, Shear rate area under curve. * vs. −10 (BL), p < 0.05 and ^†^ vs. 110 min of LPP.

### Oxidative Stress to Postprandial Lipemia Plus Disturbed Blood Flow

**FIGURE 5:**
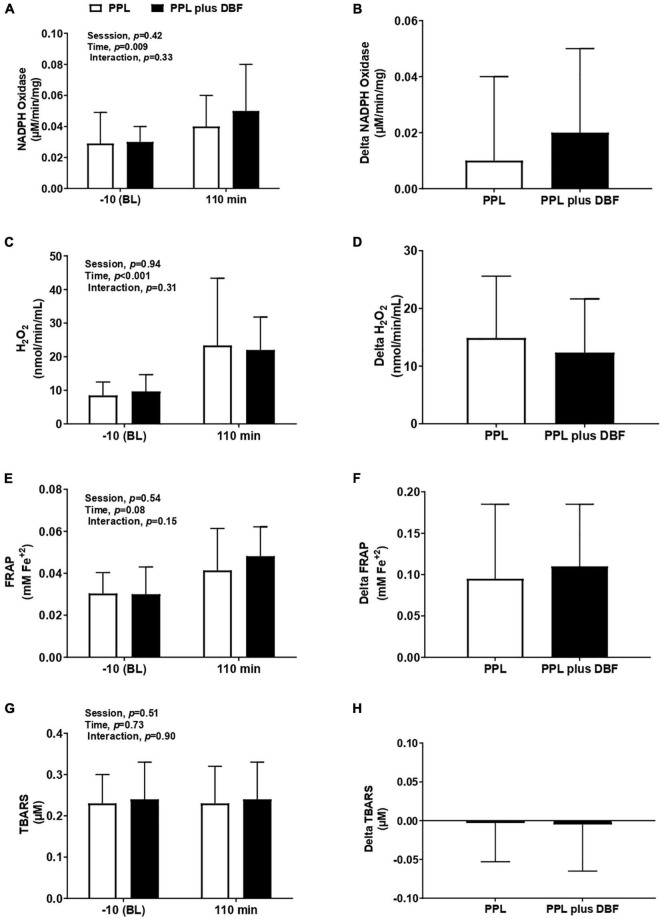
NADPH oxidase **(A)**, delta NADPH oxidase **(B)**, H_2_O_2_ **(C)**, delta H_2_O_2_ **(D)**, FRAP **(E)**, delta FRAP **(F)**, TBARS **(G)**, and delta TBARS **(H)** to postprandial lipemia (PPL) and PPL plus disturbed blood flow (DBF) in healthy individuals. Nicotinamide Adenine Dinucleotide Phosphate Oxidase, NADPH oxidase, H_2_O_2_, hydrogen peroxide; FRAP, ferric reducing/antioxidant power; TBARS, thiobarbituric acid reactive substances. Data presented as mean ± standard deviation. Absolute delta was calculated as 110 min variable – baseline variable.

Oxidative stress was evaluated by oxidant (NADPH oxidase and H_2_O_2_) and antioxidant substances (FRAP), and lipoperoxidation (TBARS). PPL and PPL plus DBF similarly increased NADPH oxidase (time effect, *p* < 0.009, Figure 5A) and H_2_O_2_ (time effect, *p* < 0.01, Figure 5C). Delta analyses revealed that NADPH oxidase response tended to be greater in PPL plus DBF than PPL (*p* = 0.07, Figure 5B), but H_2_O_2_ response was not different between the sessions (*p* = 0.33, Figure 5D). No changes were observed to FRAP (*p* > 0.05, Figures 5E,F).

To lipoperoxidation, both PPL and PPL plus DBF did not alter circulating TBARS (interaction effect *p* = 0.90, Figure 5G). Also, there were no differences in TBARS response (*p* = 0.90, Figure 5H) between sessions.

### Endothelial Responses to Postprandial Lipemia Plus Disturbed Blood Flow

**FIGURE 6:**
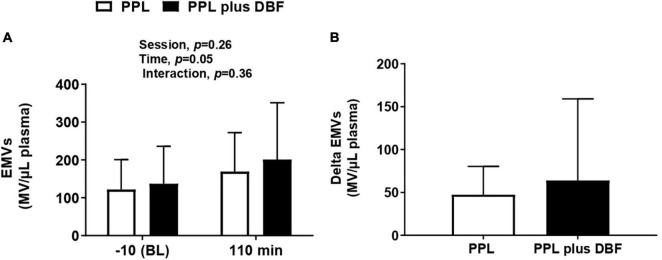
Endothelial microvesicles [**(A)**, EMVs] and changes **(B)** to postprandial lipemia (PPL) and PPL plus disturbed blood flow (DBF) in healthy individuals. Data presented as mean ± standard deviation. Absolute change (delta) was calculated by subtracting values at 110 min from baseline.

To investigate whether fat load and blood flow disturbances induce endothelial damage, circulating EMV levels were measured during experimental sessions. PPL plus DBF and PPL significantly increased circulating EMV levels (time effect, *p* = 0.05, Figure 6A). Delta analysis revealed that EMV responses were not different between sessions (*p* > 0.05, Figure 6B).

## Discussion

The main findings of the present study revealed that PPL plus DBF had an additive effect on the reduction of the brachial artery flow-mediated dilation in healthy subjects. In addition, PPL plus DBF and PPL similarly increased the circulating EMV levels, NADPH oxidase, and hydrogen peroxide. However, no changes were observed in FRAP and lipoperoxidation.

In recent years, the importance of hemodynamic forces on endothelial function and metabolic variables has become more and more evident (Silacci et al., 2000). Several studies have demonstrated that endothelial cells exhibit a capacity to discern between different hemodynamic environments (Gimbrone et al., 2000; García-Cardeña et al., 2001). Experimental studies in hypercholesteremic animals show that the association of hyperlipemia and oscillatory flow leads to endothelial dysfunction, accelerating the development of atherosclerotic disease (Koskinas et al., 2013; Pedrigi et al., 2015). It has been reported in humans that both PPL and DBF cause endothelial damage, but whether the combination of these factors has an additive effect on the loss of endothelial function has not been described so far. We found that a single acute episode of PPL plus DBF evoked a greater reduction in brachial artery endothelial function at 110 min after exposure to the stimulus, suggesting an additive effect. Because most individuals in Western society are in a postprandial state most of the time (Sies et al., 2005), routine exposure to PPL plus blood flow disturbance associated endothelial dysfunction may significantly affect the development of atherosclerotic cardiovascular disease.

Hyperlipidemia and blood flow disturbances can affect endothelial function in different ways. Increased oxidative stress seems to play an important role. Of note, we observed an increase in some oxidant enzymes like NADPH oxidase and H_2_O_2_. However, this increase was similar between sessions and, therefore, does not explain the greater reduction in endothelial function observed in PPL plus DBF compared with PPL. Another mechanism that could explain this response would be increase in TBARS, but both PPL plus DBF and PPL did not change in this variable. There is no definitive explanation for a high reduction in endothelial function that PPL plus DBF induced in our study. It is possible that this response is associated with an imbalance between vasoconstrictor and vasodilator substances. In fact, it has been shown that fat overload or blood flow disturbances decreases NO bioavailability, and increases endothelin-1 and thromboxane A_2_ (Lind, 2002; Wang et al., 2002; Hsu et al., 2019). Furthermore, transient damage to the glycocalyx can also occur (Machin et al., 2019). This luminal surface layer on the vascular endothelium presents mechanical stress shear receptors (mechanoreceptors) to stimulate NO release. Therefore, damage to the glycocalyx can compromise endothelial function *via* inhibition of the NO release.

EMVs are small membrane vesicles (< 1.0 μm) released into the circulation in response to injury, activation, and apoptosis (Dignat-George and Boulanger, 2011; Schiro et al., 2014), then, they have been used as a systemic marker of endothelial phenotype (Lugo-Gavidia et al., 2021). Previous evidence has demonstrated an association of fat overload or DBF and increases in circulating EMV levels. Indeed, in the present study, we found that both LPP and LPP plus DBF evoked a significant increase in EMVs. However, differently from what we previously hypothesized DBF did not aggravate the MVE responses to PPL. It is possible that the EMV increase was not greater in the LPP session because the vascular insults caused by these stimuli were acute, suggesting that possible differences between them should be more evident in conditions of chronic stimulation.

Some limitations are present in our study. First, the study had a small number of participants (*n* = 18), and all were healthy. This does not allow us to say whether our results can be inferred for other populations, especially those who are at increased cardiovascular risk (e.g., patients with hypercholesterolemia, diabetes, obesity, or metabolic syndrome). Second, we did not evaluate the endothelium-independent function due to the prolonged effect of nitroglycerin on the vascular system and that repeated measures of endothelium-independent dilatation could compromise the experimental protocol. However, previous evidence has documented that acute metabolic changes or flow profile disturbances do not affect vascular smooth muscle reactivity in healthy subjects (Lundman et al., 2001; Thijssen et al., 2009). Third, the study did not include a control session, without stimulus (PPL or DPF). This does not allow us to say whether the observed vascular insults did not occur over time. However, pilot data from our laboratory (unpublished data) revealed that in a control condition (no intervention), the time (effect of time) does not alter endothelial function. Fourth, we evaluated the impact of disturbed blood flow in brachial artery endothelial function, a vascular bed that is not commonly associated with atherosclerosis-related complications. But previous evidence has shown that the effects of retrograde shear in the brachial artery can be extrapolates to other atherosclerotic-prone arteries (e.g., femoral artery) (Schreuder et al., 2014). Finally, we did not measure other mediators (e.g., NO and pro-inflammatory profile) that could provide more information about mechanisms to explain our findings.

## Conclusion

Postprandial lipidemia (PPL) associated with DBF has an acute additive effect on the reduction of brachial artery endothelium-dependent function 110 min after stimuli in healthy individuals, despite a similar increase in NADPH oxidase and hydrogen peroxide. PPL plus DPF and PPL did not modify plasma levels of TBARS and antioxidant defense, assessed by FRAP. Finally, PPL plus DBF and PPL similarly increased circulating EMVs. Further studies are needed to understand the mechanisms associated with endothelial dysfunction induced by the association of fat overload and blood flow disturbances and to propose preventive strategies (e.g., physical exercise or antioxidant supplementation) against these vascular insults.

## Data Availability Statement

The original contributions presented in the study are included in the article/supplementary material, further inquiries can be directed to the corresponding author.

## Ethics Statement

The studies involving human participants were reviewed and approved by the Research Committee of the Heart Institute of Clinics Hospital of the University of São Paulo Medical School (CAAE 71981517.8.0000.0068). The patients/participants provided their written informed consent to participate in this study.

## Author Contributions

GA and TS conceived experimental designs, performed experiments, interpreted results, and drafted the manuscript. HR and NR conceived experimental designs, interpreted results, and edited the manuscript. DF, JI, AD, and AA performed experiments and edited the manuscript. FMC-C and KA interpreted results and reviewed and edited the manuscript. MI and AS conceived and designed the study, interpreted results, and reviewed and edited the manuscript. All authors contributed to the article and approved the submitted version.

## Conflict of Interest

The authors declare that the research was conducted in the absence of any commercial or financial relationships that could be construed as a potential conflict of interest.

## Publisher’s Note

All claims expressed in this article are solely those of the authors and do not necessarily represent those of their affiliated organizations, or those of the publisher, the editors and the reviewers. Any product that may be evaluated in this article, or claim that may be made by its manufacturer, is not guaranteed or endorsed by the publisher.
